# Environmental Hazards and Glial Brain Tumors: Association or Causation?

**DOI:** 10.3390/ijms26157425

**Published:** 2025-08-01

**Authors:** Robert P. Ostrowski, Albert Acewicz, Zhaohui He, Emanuela B. Pucko, Jakub Godlewski

**Affiliations:** 1 Department of Neurooncology, Mossakowski Medical Research Institute, Polish Academy of Sciences (MMRI-PAS), 02-106 Warsaw, Poland; 2 Department of Neuropathology, Institute of Psychiatry and Neurology, 02-957 Warsaw, Poland; 3 Department of Neurosurgery, The 1st Affiliated Hospital of Chongqing Medical University, The Friendship Road of Yu Zhong District, Chongqing 400016, China

**Keywords:** glioma, glioblastoma, risk of glioma, environmental hazards, oxidative stress

## Abstract

Progress in establishing environmental risk factors and, consequently, prophylactic measures for glial tumors, particularly for glioblastomas, is of utmost importance, considering the dismal prognosis and limited treatment options. This report surveyed updates on established and recently identified factors that can predispose a patient to glioma formation while highlighting possible mechanistic links and further research directions. In addition to established factors that increase the risk of glioma, i.e., brain irradiation and several genetic syndromes, another group consists of likely factors contributing to such risks, such as the use of tobacco and those yielding ambiguous results (e.g., UV exposure). Oxidative stress is a common denominator for several types of exposure, and a mechanistic background for other factors remains elusive. Nevertheless, the analysis of clinical and basic research strongly suggests that, apart from the effect of environmental stressors on DNA alterations and mutation burden, the impact of modifying the tumor microenvironment should be considered. Identifying the involvement of environmental hazards in gliomagenesis and glial tumor progression would lower overall risk by modifying clinical practice, patient management, and lifestyle choices. Further verifying the environmental hazards in glioma formation and progression would have far-reaching implications for neurologists, neurosurgeons, and patients.

## 1. Introduction

Brain tumor diagnosis is devastating news, especially if high-grade gliomas are involved, such as in glioblastoma. These gliomas almost universally indicate a patient’s demise within a matter of months [1,2,3,4]. Glioma is a collective term denoting neoplasms of glial cell lineage. As a broad term, it comprises neoplasms expressing markers of different types of glia, including astroglia, oligodendroglia, and ependymal glia. The exact identity of the cell population that gives rise to gliomas of a particular type is challenging to pinpoint and still controversial. The existing evidence, however, points to precursor cells in the subventricular zone, oligodendroglial precursors, and subependymal pluripotent cells as the cells of origin for astrocytomas, oligodendrogliomas, and ependymomas, respectively [5]. Gliomas have been WHO-classified as grades I through IV, reflecting their increasing degree of malignancy. Low-grade gliomas (grades I and II) can be characterized by slow growth and mild invasiveness. Grades III and IV are termed malignant gliomas, showing rapid and invasive growth, and they may even metastasize outside the cranium sporadically. The gliomas of the astrocytic lineage group are formed by astrocytomas, that can represent all WHO grades (I–IV), and glioblastoma, which has been assigned to WHO grade IV. The primary diagnostic distinctions between grade IV astrocytoma and glioblastoma (also grade IV) are based on their genetic makeup. In principle, grade IV astrocytomas almost invariably carry IDH1/2 mutations, while glioblastoma is always IDH wild type; secondly, the presence of either TERT promoter mutation or EGFR gene amplification, as well as chromosome 7 gain along with chromosome 10 loss qualify astrocytic gliomas as a glioblastoma, even when some histological markers of malignancy are absent.

On the other hand, homozygous CDKN2A/B deletion in diffuse IDH-mutant astrocytomas is a sufficient prerequisite for assigning it to grade IV, irrespective of the presence or absence of malignant histology [6]. Nonetheless, despite equivalent grades, glioblastoma carries a worse prognosis than an IDH-mutant malignant astrocytoma. For the latter, the median overall survival is 10 years when associated with grade III and 3 years with grade IV [7]. Although 15 months is often cited as the overall mean survival for glioblastomas, analyses from several medical centers yield an average survival of less than a year from diagnosis [8,9,10]. Thus, gliomas are classified according to their cells of origin, level of histological aggressiveness, and molecular background, all associated with more or less favorable prognosis.

This manuscript mainly deals with the impact of environmental hazards on the formation of gliomas with astrocytic lineage (astrocytic gliomas), with a particular emphasis on glioblastomas.

Well-established factors that increase the risk of developing gliomas include ionizing radiation that most often results from medical procedure-related exposures, as well as several genetic syndromes, including Li–Fraumeni syndrome, Turcot syndrome, and neurofibromatosis type 1 [11,12,13]. In several countries, the incidence of high-grade gliomas is increasing, especially in children, adolescents, and older people, thus suggesting environmental or lifestyle factors are at play [14,15,16]. Moreover, in recent years, neoplasm incidence data have likely been underreported due to delays in data collection caused by the COVID-19 pandemic [17]. Nevertheless, the incidence of malignant brain tumors in some regions is projected to increase by nearly 40% in the fourth decade of the 21st century [18,19]. Over the years, research has examined the environmental factors linked to brain tumorigenesis, among others, via promoting oxidative/nitrosative stress [20,21]. Putative tumor-promoting environmental stressors warrant further research, including analyzing longer exposure durations and larger sample sizes [22,23]. Overall, oxidative stress for gliomagenesis can be an appealing hypothesis as it can potentially bridge environmental hazards and the cell’s molecular machinery [24]. The consensus is that gliomas are characterized by increased oxidative stress, which is reflected by higher oxidation potential. Such oxidative stress can poise susceptible cells for malignant transformation by instigating DNA damage. In principle, reactive oxygen species (ROS) targets purine and pyrimidine bases within genomic DNA, inducing modifications that enable mutations and chromosomal alterations, thereby modifying the functions of both oncogenes and tumor suppressor genes [25,26,27]. Indeed, a high tumor mutational burden has been found in some gliomas, although the link between accumulated mutations and environmental mutagens warrants further investigation [28,29]. Moreover, ROS can activate the constituents of several pathways that participate in glioma formation, survival, and invasiveness, cell proliferation, apoptosis, and angiogenesis, including the HIF-1, EGFR, and Akt pathways [25,30,31]. Importantly, oxidative stress-responsive genes are supportive of a glial tumor immunosuppressive microenvironment and thus promote the progression of tumors, making it highly desirable to curb brain oxidative stress after diagnosis and standard-of-care therapy as a progression-preventive measure [32,33,34,35,36,37,38,39].

## 2. Literature Search

PubMed, Scopus, Web of Knowledge, and Google Scholar databases were queried using terms such as glioblastoma/glioma and given environmental factors, covering the timespan between April 1994 and March 2025. A total of 236 articles were scrutinized, including three cases of online journalism articles, two official publications from research centers at political and educational institutions (UN and Oxford University), and two electronic books.

### 2.1. Brain Irradiation

Brain exposure to ionizing radiation increases the risk of glioma formation even years later, including in areas that receive low doses. In general, the risk of arising secondary neoplasms, i.e., triggered by radiotherapy, is increased at higher doses of radiation [40,41,42]. A substantial portion (as much as 20–50%) of human exposure to ionizing radiation is derived from medical emitters, which is set to increase [43,44]. Even though DNA damage via radiation-induced ROS is a common factor in the formation of various secondary brain tumors, newly formed tumors often present with a molecular background distinct from that of the treated tumor, even within the same lineage of neoplasm, thus reflecting the action of diverse oncogenic pathways [45,46,47,48].

Interestingly, an often-overlooked environmental factor proposed to be associated with increased glioma incidence is local radon exposure. Radon, which gives off α particle radiation, has been found to increase the levels of 8-OHdG, a routine biomarker of oxidative DNA damage, in the urine and lymphocytes of laboratory animals [49]. This indicates that the health effects of radon exposure, including gliomagenesis, include an oxidative stress component. While coal mining and burning coal due to domestic cooking and heating are sources of radon, basement exposure, where radon seepage occurs, is the leading cause of radon-related morbidity. Likewise, liquid petroleum gas (LPG) and compressed natural gas (CNG) are significant sources of radon and its derivatives [50]. In all these instances, inhalation is the primary exposure route of the brain that increases the risk of radon-attributable tumors.

### 2.2. Agricultural Chemicals

Agricultural pesticide exposure is one of the significant occupational risks relevant to large cohorts. The link between it and the risk of gliomas is interestingly found amongst male but not female farmers. In addition, other farm exposures might also be responsible for the increased risk of brain cancer [51,52]. The meta-analysis and literature review indicated a 13% overall increase in the risk of brain cancer (predominately gliomas) morbidity and mortality, and it was significantly greater in association with livestock farming than crop farming [53]. Crop and livestock farming utilize many potentially hazardous chemicals to humans, especially among farm workers and their families. This reflects the high demand for chemicals to protect crops from pests and weeds. Significantly, pesticide sales have increased yearly to feed human populations and to meet farm animals’ dietary needs [54]. Chronic exposure to organophosphate pesticides, including chlorpyrifos, carbamate fungicides, and herbicides used to protect crops, increases the incidence of primary brain tumors in farmers [55,56,57,58]. The underlying mechanism has been proposed to rely on the induction of oxidative stress, thus pointing to a need to more closely biomonitor farm workers and their families for markers of oxidative/genotoxic stress [59,60,61,62,63].

Organophosphates and pyrethroids are also used in livestock farming as insecticides—veterinary treatments against parasitic infestations and biocidal products to eliminate insects on livestock premises [64,65]. While the increased risk of meningioma was associated with mixed dairy and cow farming, studies have shown that pig farming relying on pyrethroid pesticides has mainly been related to the risk of gliomas [61]. Insecticides could also be one of the driving forces behind the positive association between childhood exposure to farm animals and childhood brain tumors [66].

Likewise, household pyrethroids, including mosquito and cockroach pesticides, increase the risk of childhood tumors, including gliomas (59 out of 161 childhood brain tumor [CBT] cases), medulloblastomas (37 out of 161 CBT cases), and other cancers [67]. In particular, trans-3-(2,2-dichlorovinyl)-2,2-dimethylcyclopropane carboxylic acid (trans-DCCA) and 3-phenoxybenzoic acid (3-PBA) urine metabolites, both derived from pyrethroids, were positively associated with an increased risk of childhood brain tumors. Thus, household pesticides have been linked to childhood brain tumors; however, the environmental spillover of agricultural chemicals outside farming areas cannot be excluded [68]. The immunotoxicity and genotoxicity mechanisms of pyrethroids and their metabolites, such as 3-PBA, may involve elevated levels of ROS, as experimentally determined [69,70,71,72].

Unexpectedly, exposure to pesticides not only increases the risk of gliomas but renders glioma cells resistant to anticancer agents [73]. However, the latter topic remains largely unexplored.

### 2.3. Electromagnetic Fields

An increased risk of brain cancer was reported among military personnel exposed to microwave/radiofrequency radiation [74]. The increased vulnerability risk of organs in the abdominal cavity and the head to radar waves was also determined [75]. Military personnel with occupational exposure to radiofrequency radiation (RFR) are at risk of developing hematolymphoid neoplasms, possibly due to the formation of ROS, among others [76,77]. Of note, microwave exposure can induce both oxidative and nitrosative stress in the brain, the latter signifying an imbalance in generating and eliminating reactive nitrogen species capable of inducing DNA damage [78,79]. A recent study found that ground personnel in the U.S. Air Force have a 19% higher rate of developing cancers of the brain and nervous system than the general U.S. population [80].

Further studies are needed to determine whether military sources of RFR may contribute to such elevated rates. It has been discovered that there are higher morbidity rates due to brain tumors in radiofrequency- and microwave-exposed military personnel vs. non-exposed individuals [81]. All this information may stress the importance of using radar absorption materials in human exposure mitigation aside from their primary purpose, which is delivering stealth properties [82,83].

Microwave radiation within the range of cell phones (0.9–2.45 GHz) has also come under scrutiny. Animal experiments were performed on a cohort of 2448 rats exposed to a 1.8 GHz GSM emitter device 19 h/day for their total life span. The study found an increased incidence of heart schwannomas in male rats (at 50 V/m; SAR of 0.1 W/Kg). The electromagnetic radiation dose-dependent increase in the incidence of malignant glial tumors was observed in treated female rats (up to 1.5% vs. 0.5% in controls), although it did not reach statistical significance [84]. Microwaves within the GSM frequency band increased lipid peroxidation and DNA single-strand breaks in brain cells of experimental animals [85,86]. As unrepaired DNA strand breaks are widely regarded as a predisposing factor for tumor formation across many biological systems [87,88,89,90], mobile phone exposure has been classified as a possible human carcinogen by the International Agency for Research on Cancer (IARC) [91]. Among persons who have held cellular phones to their ears for many years, an increased risk was found for gliomas, although they were mainly low-grade gliomas [92,93]. Thus, a safer use of mobile devices has been recommended, including more frequent messaging and using the speaker mode or a headset when making phone calls [94].

However, data supporting the link between cell phone use and gliomas is elusive, at best. For a long time, since the inception of the widespread use of cell phones in the 1990s, trends in brain cancer incidence have not mirrored the substantial increase in the technology’s use [91]. There is a possibility that cannot be excluded, namely that the effect is taking a long time to manifest; thus, it remains to be seen whether cell phone use is significant. Moreover, the advancing age of exposed individuals may accelerate the gliomagenic effect in the future. The exploding number of technology users, which doubled only as recently as between 2015 and 2020 [95], will introduce an unprecedented volume of potentially affected individuals. Therefore, even small-scale effects are poised to be revealed in the future.

### 2.4. Diet and Lifestyle

As nutritional habits and other lifestyle choices that depend on the social environment can affect the overall incidence of cancer, they are briefly touched upon in this review. For instance, it is known that high-lipid and high-carbohydrate diets enhance glioma aggressiveness [96]. Thus, the question arises as to whether an unhealthy diet can induce gliomas. Retrospective analysis-based studies from the U.S. and Canada have largely failed to demonstrate the association between different groups of nutrients and glioma risk [97]. However, data from particular regions have reported noticeable findings in this respect; for example, one study found that dark yellow vegetables, such as carrots, and dietary fiber intake were associated with a reduced risk of gliomas [98]. Similarly, more recent comprehensive analyses comprising 7278 glioma cases across the globe concluded that a high intake of fruits and vegetables reduces the risk of gliomas [99]. Arguably, an unbalanced and unhealthy diet rather than a particular nutrient may contribute to its occurrence, while vegetarian products seem to reduce the risk [100].

Vegetables and fruits are rich sources of carotenoids and antioxidants, indirectly pointing towards a free radical background of glioma formation. Similarly, a vitamin A-and B-rich diet in the Chinese population seems to reduce the risk of gliomas, along with a high dietary intake of magnesium, calcium, zinc, and copper [101,102,103]. Several studies have suggested that tea and coffee consumption, especially when combined, reduces the risk of gliomas in American, Asian, and European populations [104,105,106]. Moreover, there seems to be a geographic diversity in the most effective beverage, as coffee consumption was associated with a lower incidence of gliomas in Asian populations. By contrast, the most reduced risk of gliomas in the American population was detected amongst tea drinkers [107].

Food and nutrition disorders may also contribute to primary brain tumor morbidity. However, the link between BMI and the risk of glioblastoma remains unclear. Obesity among teenagers has been linked to glioma incidence when determined by using pooled studies. At the same time, one multicenter prospective study derived an inverse correlation between BMI and glioblastoma risk [108,109]. Interestingly, a Norwegian study found a positive correlation between the risk of glioblastoma NOS (not otherwise specified) and height [110]. A tendency towards a higher BMI in short-stature people could align with such findings [111]. However, several studies have failed to prove a trend toward the increasing risk of gliomas and glioblastomas with increasing BMI [112,113]; others (e.g., the authors of a population-based cohort study of Koreans) have argued that the analysis of abdominal obesity, rather than merely BMI, can yield a positive association with the risk of glioma development [114]. This underscores the need for further research in this area, inviting the audience to contribute to ongoing scientific exploration. Recently, one research group implied that the total-cholesterol-to-total-lipid ratio in large VLDL and the ratio of polyunsaturated fatty acids to total fatty acids (PUFAbyFA) are risk factors for gliomas, while free cholesterol or phospholipids in HDL are protective against gliomas based on an analysis of publicly available genome-wide association study data [115]. The ratio of polyunsaturated fatty acids to total fatty acids is a critical indicator of lipid metabolism within biological organisms, and can be associated with neuroinflammation and enhanced tumor growth, especially owing to the impact of omega-6 polyunsaturated fatty acid [116]. These results, if further substantiated, may require establishing new comprehensive recommendations on brain tumor prevention alongside preventing cardiovascular and cerebral vascular diseases.

### 2.5. Impact of Combustion Products

The combustion products coming from military and civilian burn pits and combustion vehicles (especially ultrafine particles: PM < 100 nm in diameter) have been suspected of causing the genetic alterations underlying gliomagenesis, but the evidence needs to be further substantiated, as the cause-and-effect relationship may depend on a still unaccounted-for genetic background [117,118,119]. Particularly worrisome is the fact that maternal or paternal occupational exposure to diesel exhaust (incl. motor vehicles) may increase the risk of childhood brain tumors, including low- and high-grade gliomas [120]. Although, in 2012, the IARS classed diesel exhaust as a carcinogen concerning lung cancer and possibly bladder cancer; in more recent years, the link between ultrafine (<100 nm) particles present in both diesel and gasoline exhausts and brain tumor incidence was revealed by a study by Weichenthal et al. and has been confirmed by other research groups [118,119,121,122,123]. Convincingly, air pollution can lead to proinflammatory activation and oxidative stress in glial cells, although more reliable models for researching these effects are needed [124]. Another source of combustion products consists of kerosene stoves in underprivileged housing, as kerosene carries a substantial association with astrocytomas in children [125]. These observations are consistent with the demonstrated association between a higher incidence of gliomas in areas with airport operation activity [126], which can be linked to the effect of jet fuel (basically kerosene-based) combustion products on glioma formation.

In addition, there might be a link between exposure to the chemical agents used in firefighting and the risk of developing gliomas. Haloalkanes are widely used in flame retardants and fire extinguishants. Mutational signatures associated with haloalkane exposure were found in glioma samples collected from firefighters, who were participants in the University of California San Francisco Adult Glioma Study (of note, single-base substitution signature 42). Moreover, increased glioma risk was noted in firefighters from the same study [127]. There was also a positive correlation between the median number of variants in the samples attributable to SBS42 and firefighting years. Of note, SBS42 is a single-base substitution mutational signature 42 observed in glioma tumors and is primarily associated with occupational exposure to haloalkanes [127]. Taken together, these data compellingly point to exogenous mutagenesis being involved in glioma inception. Therefore, for mutagenic compounds associated with occupational exposures, the patterns of damaged/mutational signatures need to be established while investigating the potential drivers of glioma phenotypes.

### 2.6. Ultraviolet

UVA (315–400 nm) and UVB (280–315 nm) are capable of inducing DNA damage that may lead to glioma formation, while UVC (100–280 nm wavelength) has been used as a potential adjuvant treatment to induce glioma cell apoptosis, preferably delivered locally by implantable systems [128]. The question remains as to whether sources of UV light may increase the risk of brain cancer in addition to cutaneous neoplasms. Occupational exposure to UV light seemed to increase the risk of gliomas in Australian men, although it could be confounded by other exposures/activities associated with high UV [129]. Regarding UV light and the risk of gliomas, the exact cause–effect relationship remains elusive, unlike in skin cancer. On the other hand, ultraviolet B is required to synthesize vitamin D from 7-dehydrocholesterol in the skin. Thus, intense solar operation and other UV sources may be perceived as a double-edged sword, as they cause DNA damage but might protect against the onset of gliomas via an endogenous boost of steroids with antiproliferative activity [130].

### 2.7. Heavy Metals

Environmental exposure to heavy metals (density > 5.0 g/cm^3^), including cadmium, chromium, copper, lead, mercury, nickel, and arsenic (metalloid), can cause DNA damage acting via oxidative stress and induce multiple epigenetic alterations [131,132,133]. Lead and cadmium, known for causing significant oxidative DNA damage, have been linked to an increased incidence of gliomas in industrial workers, although the evidence has been inconsistent [134,135,136,137]. Prolonged exposure to cadmium leads to a decrease in intracellular glutathione concentration and, consequently, an increase in the extent of oxidative DNA lesions in cells [138]. Inconsistent concentrations of heavy metals found in glioma and non-glioma samples did not support causative conclusions [139]. Yet, other reports have pinpointed elevated contents of manganese and lead not only in cancerous tissues but in the serum, cell fraction, and CSF of patients with malignant brain tumors, including glioblastomas (54 out of 61 analyzed tumors in total). Thus, further studies, not limited to metal industry workers but including larger populations, e.g., inhabitants or employees of brownfield sites, are required to shed more light on the issue. In addition, such studies should include farm workers, people working in dental care facilities, users of combustion stoves, drug users, and tobacco smokers, among others, who with their daily routines, face an increased risk of contact with heavy metals [140,141,142,143,144,145]. Thus, for the time being, the interim conclusions may state that heavy metals are likely to impact brain tumor formation but have to be further examined for such an impact.

### 2.8. Geography, Demography, and Environmental Cues

Several factors, such as geographical location, climate, and demographics, also seem to have an impact on brain tumor occurrence. Glioblastoma development rates may depend on the geographic region, although this should instead be analyzed in conjunction with the specific environmental and socioeconomic factors attributed to a given location. For example, the incidence rates of brain cancer were elevated at higher latitudes consistent with the low solar UVB exposure that leads to lower concentrations of serum 25(OH)D, as determined based on data from 175 countries [146], thus supporting the hypothesis of vitamin D as an inhibitor of glioblastoma cell growth [147]. On the other hand, Xu et al. found the highest incidence of glioblastomas among patients from the south of the U.S., a region known for a substantial level of insolation [148]. These discrepancies are possibly attributable to factors that may surpass the effect of climate change, such as diverse socioeconomic status or access to health education. This underscores the potential impact of our research on public health, making the audience feel the significance of their work in reducing the incidence of gliomas.

Environmental cues have also been implicated to be responsible for tumor initiation in the case of optic gliomas [138]. Individuals with NF1 are affected by optic gliomas (OPGs) at a rate of 15–20%, while the mean age at OPG diagnosis is 4.5 years [149,150]. A series of elegant experiments have revealed that optic nerve gliomas were not developed or developed as small and less proliferative in NF1-OPG mice that were reared in the dark during time periods typical for tumor initiation and growth [151]. In addition, mice engineered to develop the blue light-sensitive channel rhodopsin (ChR2) in ganglionic cells in response to blue light exposure developed large, highly proliferative glial tumors [151]. Thus, the light-induced activity of the optic nerve appears to contribute to glioma initiation in this particular scenario. The exact interplay of the bi-allelic inactivation of the NF1 tumor suppressor gene and light exposure leading to tumor initiation requires further study, although the authors have experimentally implicated neuronal ADAM10 sheddase (acting on neuroligin-3) secreted into tumor microenvironment as participating in the process. Other studies have pointed out that glioma growth can be mediated by neuronal activity-dependent cell membrane/extracellular matrix potassium fluxes, resulting in glioma cell membrane depolarization or electrochemical communications via AMPA receptor-dependent neuron–glioma synapses [152]. However, a broader conclusion may emerge from these and other studies, namely that the positive impact of environmental stressors on glioma growth can be mediated by cells from areas other than glial compartments of the CNS [150,153]. Moreover, there might be an interaction of the genetic background with environmental stimuli for tumor formation [151].

Another putative impact of light on gliomagenesis can be conveyed by the light emitted by electronic devices. It seems plausible that their light–dark cycles, misaligned with circadian rhythms, can lead to circadian rhythm sleep–wake disorders and disturb the function of the glymphatic system, thereby producing a pro-tumor effect [154,155]. The glymphatic system is the pathway facilitating the clearance of waste products through the brain parenchyma via the perivascular space, and its dysfunction has been implicated in the progression of brain tumors and a reduced efficacy of therapeutic interventions [156]. Establishing the consequences of exposure to electronic screens in this respect, as well as providing safety recommendations, requires further studies.

Data from countries in different geographical locations can consistently provide clues as to the incidence of gliomas, highlighting the global impact of this research. In the northern hemisphere, the two countries with the highest brain cancer incidence (per 100,000, ages ≥ 15 y), Croatia (12.0 for males; 9.4 for females) and Poland (9.8—M; 9.2—F), have similar ratios of urban populations (60% and 57.6%, respectively) [157,158].

Although these countries are very different in many ways, the urban/rural dichotomy appears to shape the differences in the distribution and impact of global glioma risks. A rural–urban poverty gap exists in most countries worldwide, and rural poverty tends to exceed urban poverty [159,160,161].

More than 3.1 billion people worldwide—or 42 percent—could not afford a healthy diet in 2021, an indication of living in poverty, most often in rural habitats [162]. Also, smoking prevalence is higher in rural communities [163]. Moreover, rural regions are strongly associated with increased exposure to agriculture chemicals. Especially when analyzing combined exposure to fertilizers, herbicides, insecticides, and fungicides, the risk of developing gliomas becomes statistically significant [164].

On the other hand, excessive mobile phone or broadband use is more widespread in urban populations, as is the prevalence of viral infections linked to gliomagenesis [165,166].

However, the differences in glioma incidence between rural and urban communities are not uniformly shaped. For example, for Asians, Pacific Islanders, American Indians, and Alaska natives, a higher incidence of gliomas was observed in rural areas. By contrast, amongst white individuals, higher glioma incidence was observed in urban communities with high socioeconomic status [167]. Not surprisingly, many studies have reported a positive correlation between high socioeconomic status and improved survival, occasionally the lack thereof, but never the opposite [168,169]. In a study involving patients identified via the Swedish National Quality Registry for Brain Tumors, a low education level was associated with reduced survival for patients with WHO grade III and IV gliomas in multivariable survival analyses, but no differences in survival were found in relation to travel time, cohabitation status, or region of residence [170]. The analysis of the above data may lead to the conclusion that various ethnicities are differentially susceptible to particular environmental hazards linked to glioma formation. By contrast, the prognosis is somewhat improved with improved access to healthcare.

### 2.9. Infectious Diseases

Infectious diseases have long been implicated in the development of cancers, including brain tumors, with a particular emphasis on double-stranded DNA viruses that can directly integrate into the human genome. There is an established, yet not ironclad, link between gliomas and viral infections (JC virus [JCV; John Cunningham virus], BK virus [BKV; virus isolated from a Sudanese patient with initials B.K.], and cytomegalovirus [CMV]). This is because viral genetic material is routinely detected in tumor tissues [171,172], as is the case for JCV, which was isolated from glial tumor samples [171], where the interference of the virus with the host cell machinery was postulated as the mechanism of malignant transformation [173]. Similarly, laboratory animals inoculated with BKV developed gliomas, amongst other neoplasms [174].

Other pathogenic viral species linked to glioma formation include Epstein–Barr virus (EBV), human herpesvirus 6 (HHV6), human papillomavirus (HPV), and simian virus 40 (SV40). Epstein–Barr virus (EBV or HHV-4), one of the most widespread human viruses (up to 95% of the population infected), has been implicated in gliomagenesis due to enhanced malignancy following viral infection, in addition to infectious mononucleosis, Burkitt’s lymphoma, and primary CNS lymphomas [175]. Researchers reported that 21.4% of brain tissue from glioma patients, but none of the control group tested positive for EBV DNA [176]. EBV-positive glioblastoma cases tend to be associated with more aggressive tumors and poorer survival [177]. The hypothesized mechanism of malignant transformation induced by EBV in the glial tissue involves cellular receptors for EBV present in several types of glial cells [178]. Interestingly, the EBV genome encodes microRNAs that target host–cell transcripts, thus inhibiting apoptosis and promoting tumor growth [179].

Integrating HHV-6 into chromosomes contributes to familial glioma etiology by promoting cell death resistance, telomeric instability, and replicative immortality [180]. Another oncovirus with significant prognostic implications for gliomas and meningiomas is HPV [171]. One study detected HPV in 23% of glioblastoma specimens by multiple methods, suggesting that glioblastoma cells produce HPV viral proteins [181]. Likewise, SV40 likely plays a role in brain tumorigenesis, as it is associated with poor prognosis for low-grade gliomas [182]. This virus, of monkey origin, has historically spread across human populations due to contaminated anti-polio vaccines and possibly due to exposure in professional environments [183]. SV40 sequences were detected in glioblastomas with a higher prevalence than in control tissues. However, tumors induced experimentally by SV40 transgenes or intracerebral virus inoculations mainly comprised ependymomas and choroid plexus papillomas. Notably, this virus can use the host cell microRNAome to its advantage [184].

In the early 2000s, the glioblastoma field was expanded by reports demonstrating CMV gene products in tumor tissue but not normal brains. Since this initial observation, several groups have shown an onco-modulatory effect of CMV; however, a direct association between CMV infection and glioma incidence has, until recently, been lacking [185,186]. While actively replicating CMV has not been isolated from tumor samples, it promotes high-grade glioma progression in a mouse genetic model, and many CMV proteins promote cancer hallmarks in vitro [187,188]. Mechanistically, CMV promotes glioblastoma oncogenic traits via the NF-κB-dependent upregulation of the c-MET oncogenic tyrosine kinase, and pericyte recruitment that led to increased angiogenesis was apparent in the glioma mouse model [189,190]. Despite a possible etiologic role in GBM, interest has recently centered on exploiting this association to develop immunomodulatory therapies. These approaches capitalize on the prevalence of CMV in glioma tissue to offer CMV-specific immunotherapy for glioblastomas. CMV-targeting treatment bears both anti-CMV and anti-tumor effects. As the primary anti-CMV treatment, valganciclovir has demonstrated a promising survival benefit in both newly diagnosed and recurrent glioblastoma as an adjuvant therapy [191,192]. Therefore, anti-CMV therapies are worthy of further recognition and investigation.

Interestingly, researchers discovered a significant association between malaria outbreaks and reports of brain tumor incidence from 19 U.S. states [193]. As mosquitoes are well-known vectors of multiple viral diseases (caused by viruses collectively dubbed arboviruses), in addition to protist-borne malaria, some of these viruses may play a role in tumor etiology. There are presumably also unknown mosquito-transmitted viruses/microorganisms that can promote oncogenesis. Notably, many arboviruses can cross the blood–brain barrier and thus cause direct viral invasion into the brain with severe neurologic manifestations [194]. Preliminary analyses pointed to the gliomagenic potential of the RNA virus, severe acute respiratory syndrome coronavirus 2 (SARS-CoV-2) [195], but further studies are warranted to test this hypothesis. Thus, the spectrum of tumors that oncogenic viruses may entice seems quite broad. Additional research is warranted to establish their impact on glioblastoma etiology, risk, and prognosis while providing promising therapeutic targets.

### 2.10. Recreational Substances and Habits

Several reports have addressed the recreational use of various substances in terms of brain tumor formation. For instance, one study found that those who recreationally inhale solvents (e.g., glue) have a higher risk of developing grade II–IV gliomas [196].

This link has also been studied with [197] tobacco smoking, including its passive form, and the development of gliomas, especially in young people [21,198]. Interestingly, although smoking during pregnancy was associated with an increased risk for neuroblastoma, it was not associated with astrocytomas [199]. This threat should be analyzed by also considering the still widespread habit of smoking in several global subpopulations, including non-Hispanic Caucasians in the United States, who have been linked to both the highest smoking prevalence and the highest glioblastoma incidence [200,201,202]. On the other hand, the most convincing indication of the role of smoking in triggering gliomagenesis can be derived from Asian populations. After having analyzed the data from the Korean Health Insurance System cohort, one study found that cigarette smokers have a greater risk of developing malignant gliomas, especially those who smoked ≥20 cigarettes daily [203]. So far, studies have shown that cigarette smoke can induce organic neurocarcinogen production [204], as well as generate reactive oxygen species via PKC-dependent NADPH oxidase activation in glioma cells [205]. Thus, smoking is of utmost significance for developing gliomas, as ROS have been postulated to play a role in the gliomagenesis and glioma cell survival pathways [87,100,191,192,193,206,207,208].

### 2.11. Intake of Drugs

As the use of approved medications (e.g., anti-inflammatory, contraceptive) always brings about somewhat significant side effects, the epidemiology field has focused on the role of these small-molecule drugs in cancer risk. Some of these drugs, especially those designed for prolonged treatment periods, have been associated with the risk of glioma development. For example, in a national case–control study, the long-term use (>5 years) of oral contraceptives was a risk factor for the development of gliomas in women aged 15 to 49 years [209].

In treating brain diseases, the blood–brain barrier (BBB) hinders small-molecule drugs from entering into the brain parenchyma, thereby reducing efficacy. Recently, nanomedicines that enhance the BBB crossing ability have been introduced for glioma treatment [210]. They hold several advantageous features for treating diseases thanks to their tumor-targeting capabilities, long circulation, high biocompatibility [211], and feasibility of their modifications (e.g., targeted peptide modifications, protein modifications, biofilm modifications). However, nanomedicines’ brain and systemic toxicity are challenging to evaluate due to the complexity of their composition and the difficulty of their clearance [212]. For example, while some nanomedicines significantly improve treatment efficacy, the heavy metal ions they carry (iron, copper, manganese) can accumulate in the brain, which may induce gliomas [143,213]. On the other hand, the purpose of a nanomedicine-based therapy is trying to exploit the accumulation of heavy metals, such as copper oxide nanoparticles, for therapeutic purposes, including killing bacteria and cancer cells [214]. Therefore, mitigating local or systemic side effects and standardizing nanomedicines are challenges to overcome to achieve their full clinical translation potential.

## 3. Discussion

**Figure 1:**
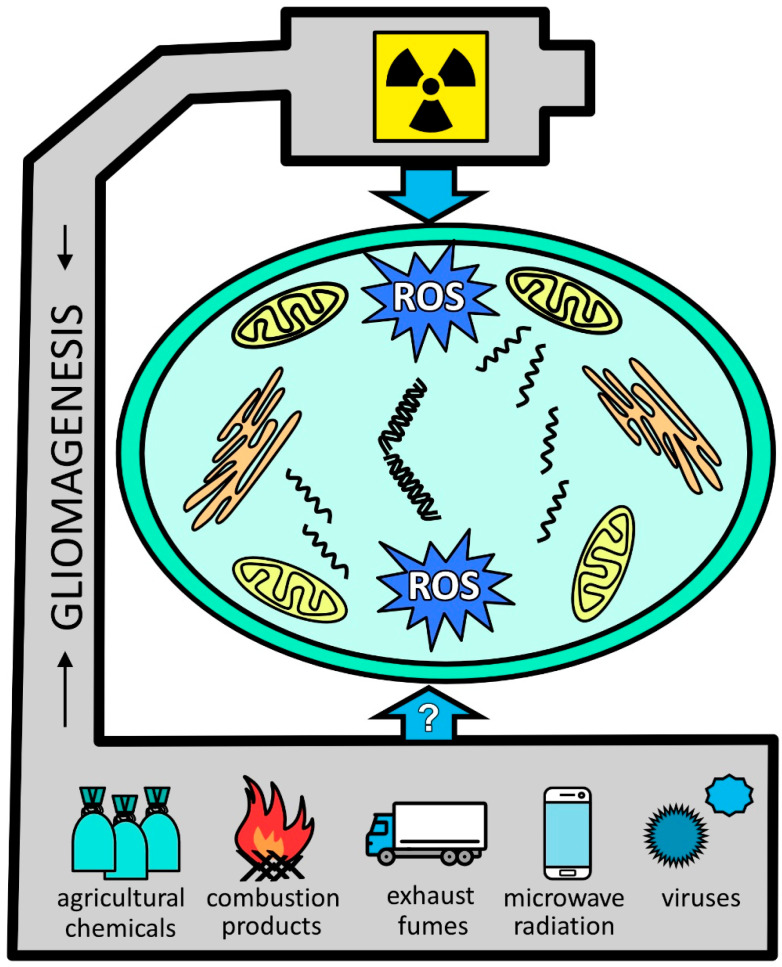
Glial tumor-promoting stressors. Ionizing radiation is among the well-documented factors leading to glioma formation and is associated with oxidative stress. With less proven effects, other factors include pesticides, burn pit-derived combustion products, and vehicle exhaust fumes. Although still somewhat speculatory, viruses are widely recognized as contributing to gliomagenesis. Abbreviations: ROS, reactive oxygen species.

Figure 1 summarizes the major environment-related factors linked to gliomagenesis. One common denominator is oxidative stress, which is incited by those stressors. However, the overall evidence remains insufficient. Not only do broader studies need to be performed but basic research would also be welcomed. Thus, Table 1 further capitalizes on the main environmental and lifestyle factors that may have an impact on glioma initiation and progression, as discussed in the present review, along with the putative underlying mechanisms and the level of evidence according to the GRADE system with respect to the cited reports [215]. In the case of some relatively newly emerged factors (e.g., cell phone use), the link between glioma incidence and those agents may appear after a more extended period.

Moreover, the impact of environmental factors on the growth of already developing glial tumors may be essential, although further studies are needed to substantiate such a notion. Parallel to those should be clinical studies of predictive and prognostic markers of gliomas associated with exposure to different environments, whether proven or suspected [216]. Patients diagnosed with gliomas who visit neurologists are not routinely checked/questioned for environmental hazards, which, if substantiated, could modify doctors’ orders/counseling to prevent further exposure and to inform public awareness of environmental hazards that would change society’s daily routines and encourage early consultation with healthcare specialists when exposure was encountered [217].

The primary concern is that the environmental hazards linked to glioma formation may affect adults and their offspring, and the underlying evidence is becoming more prevalent. Hence, preventive measures should be undertaken beforehand to protect the general population and the most vulnerable from brain tumor environmental carcinogens. Those are significant tasks for research and clinical and prophylactic medicine.

**Table 1:** Classifications of environmental factors by strength of evidence and summary of proposed mechanisms.

Environmental/Lifestyle Factors Implicated in Glioma Initiation and/or Progression	Level of Evidence	References	Proposed Mechanisms
Agricultural chemicals, pesticides	3b	[51]	Oxidative stress, DNA damage, carcinogenic organic compound formation [63]
	3a	[68]	
Brain irradiation	2a	[41]	DNA damage leading to oncogene amplifications and homozygous deletions of tumor suppressor genes [48]
Combustion products and air pollutants	2b	[118]	Oxidative stress, DNA damage [119]
	3b	[123]	
Firefighting chemicals (haloalkanes)	3b	[127]	Mutations characterized by SBS42 signature [127]
Heavy metals	2b	[135]	Oxidative stress, DNA damage, impaired DNA repair gene expression, OGG1 [133,138]
	3b	[136]	
Microwave radiation	3a	[93]	Induction of oxidative and nitrosative stress in the brain [78,79]
	3b	[92]	
	4	[81]	
	5	[218]	
Oral contraceptives	3b	[209]	Interaction of synthetic hormones with sex hormone receptors in brain cells [219,220]
Poverty	2b	[169]	Increased exposures to environmental hazards and limited access to healthcare [169]
	2b	[168]	
	2b	[170]	
Smoking tobacco	4	[198]	Increased production of neurocarcinogens and ROS [204,205]
	3a	[21]	
Viruses	3a	[186]	Activation of oncogenic pathways and angiogenesis [189,190]
	5	[185]	

Based on these findings, certain measures should be undertaken. Education on the subject of environmental hazards should include all age groups, whether in open courses or media-based cautionary and affirmative actions. More interactions between patients and occupational and environmental medicine specialists, as well as public health and general preventive medicine specialists, should be fostered. This aspect of patient care should not be neglected at any level of medical care conducted by a medical specialist. Therefore, research should continue to determine the optimal methods of monitoring environmental hazards, involving data collection and use, among other tasks, for creating community-based strategies of preventive medicine. Laboratory investigations will continue in the pursuit of establishing novel markers of environmental exposure, and possibly tailoring future therapies with respect to the environmental molecular imprints underlying the disease. It is a quite common scenario that patients discontinue their unhealthy lifestyle after knowing the unfavorable diagnosis and, even at this stage, it may offer some benefits in the course of the disease [221]. Thus, it is plausible to imply that avoiding the environmental factors underlying glioma initiation and progression may further enhance public health benefits.

This preventive aspect of medical care is viable, as it addresses the genuine needs of patients; however, it remains vastly in the strictly commercial or even gray zone of medicine. Needless to say, the public financing of disease prevention programs needs to be increased, as these programs also educate both prospective and practicing medical professionals.

### Future Research Directions

Environmental stressors have been thus far mostly studied with respect to causing mutations and genome instability, and much less regarding their impact on other established hallmarks of malignant glioma biology, including the tumor microenvironment, immune reactivity, metabolism deregulation, inflammation, and epigenetic reprogramming. Such an impact has yet to be examined. However, it is tempting to speculate that, even in patients with glioma diagnosis, the prognosis may in part be dependent upon mitigating harmful environments [222,223].

By virtue of artificial intelligence, vast clinical, molecular, pathological, and neuroimaging datasets can be analyzed in a relatively short time. Hence, it is important to develop and expand glioma registries operable in a cloud environment. In these, molecular data should be accompanied by histomorphological, clinical, occupational, and environmental data, which, when combined, could allow one to pinpoint environment–gliomagenesis inter-relations. Future multi-omics studies will examine the interplay of environmental factors with genetic predispositions [224]. However, it seems that the causal relationship between environmental hazards and gliomagenesis will only be revealed by studying the molecular signatures associated with environmental agents [225], especially because such signatures can be found in glioma precursor cells. Studies have shown that exposure to environmental agents, especially in utero, can translate to forming glioma stem cell dysregulation patterns and to acquiring cancer stem cell-like characteristics [226].

**Figure 2:**
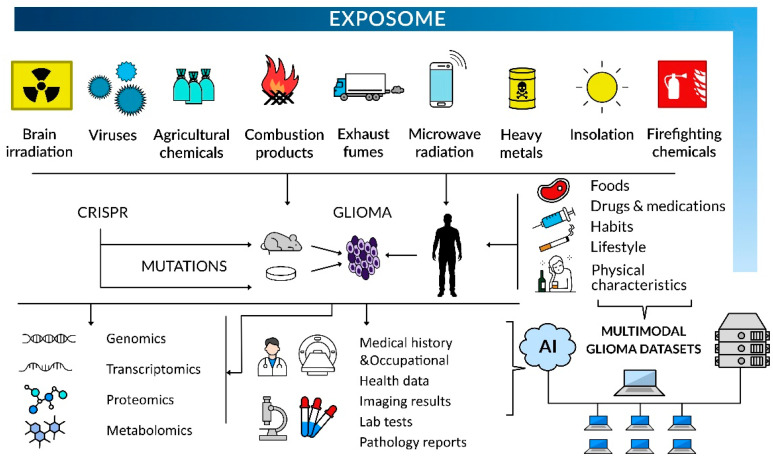
Environmental hazards can be tested in humans, laboratory animals, and cell systems, and the latter can be tested in the most controlled fashion. Upon laboratory investigations in vitro and in vivo, mutations associated with environmental stressors can be introduced with genetic engineering techniques to investigate the putative drivers of glioma phenotypes, including a multi-omics approach. Experimental systems can be subjected to artificially generated exposures to environmental mutagens, as this approach can prove causality if substantiated by demonstrating specific mutational signatures in glioma-forming cells. In glioma patients, medical history and occupational health data, imaging results, pathology reports, and lab test and multi-omics assay results should be deposited in multimodality tumor registries. The analysis of vast multimodal datasets by scientists using AI-based computing will help to pinpoint causality in glioma formation, owing to a large sample size and high power of statistical analysis.

These combined bioinformatics and molecular biology approaches will reinforce the epidemiological data analyses to identify modifiable risk factors of glioma inception and tumor development associated with occupational or environmental exposure. The concept of the exposome as a totality of exposures that an individual encounters throughout life can be applied to personalize treatment options and prognosis (Figure 2). No less important is the population-centered exposomics based on community-level monitoring of environmental exposures, including pollutants, internal markers of exposure, and population health status [227]. Progress in this respect comprises developing new protocols/procedures to investigate the exposome, e.g., organic pollutants in normal and tumoral brain tissues and establishing new networks of sensors for multiple exposures, in order to create, among others, exposomic maps [227,228]. It also involves elaborating exposome atlases and other exposomic databases to provide a framework for exposome assessment [229].

Additionally, growing cells, organoids, or breeding laboratory animals exposed to putative gliomagenic factors or engineering environmental agent-induced mutations into laboratory models might shed new light on their role in the origin of gliomagenic transformation (via the activation of oncogenic programs) and the formation of brain tumors of glial origin [230,231]. In vitro experiments can prove causality between exposure to a certain environmental mutagen and a mutational signature observed in the genomes of healthy and/or tumor cells [28]. Last, but not least, the identification of environmental epimutagens that play a role in gliomagenesis awaits further study [232,233].

Different environmental exposure molecular signatures in glioblastoma can have a diverse prognostic value; hence, their analysis could be of help with tailoring novel anti-glioma therapies. However, it seems that brain surgery, especially novel minimally invasive techniques, will still hold strong amongst glioblastoma therapies, since its cytoreductive approach can be combined with non-surgical innovative treatments to reduce tumor lysis syndrome and cytokine storm risk [234,235,236].

Nevertheless, the preventive approach to brain glial tumors based on the results of the aforementioned future molecular studies and exposome analyses will hopefully gain new momentum.

## 4. Conclusions

The causal association between most environmental hazards and gliomagenesis has not been established. However, mounting evidence suggests such an association. Novel research avenues involving epidemiology, clinical studies, and basic mechanism investigations are warranted to verify environmental hazards in glioma formation and progression, with far-reaching implications for doctors and patients alike.

## Data Availability

No new data were created or analyzed in this study. Data sharing is not applicable to this article.
